# Hot Deformation Treatment of Grain-Modified Mg–Li Alloy

**DOI:** 10.3390/ma13204557

**Published:** 2020-10-14

**Authors:** Mariusz Król, Przemysław Snopiński, Marek Pagáč, Jiří Hajnyš, Jana Petrů

**Affiliations:** 1 Department of Engineering Materials and Biomaterials, Faculty of Mechanical Engineering, Silesian University of Technology, 44-100 Gliwice, Poland; przemyslaw.snopinski@polsl.pl; 2 Center of 3D Printing Protolab, Department of Machining, Assembly and Engineering Technology, Faculty of Mechanical Engineering, VSB Technical University Ostrava, 17. listopadu 2172/15, 708 00 Ostrava-Poruba, Czech Republic; marek.pagac@vsb.cz (M.P.); jiri.hajnys@vsb.cz (J.H.); jana.petru@vsb.cz (J.P.)

**Keywords:** magnesium alloy, hot compression test, flow stress, microstructure evolution, constitutive model, processing map

## Abstract

In this work, a systematic analysis of the hot deformation mechanism and a microstructure characterization of an as-cast single *α*-phase Mg–4.5 Li–1.5 Al alloy modified with 0.2% TiB addition, as a grain refiner, is presented. The optimized constitutive model and hot working terms of the Mg–Li alloy were also determined. The hot compression procedure of the Mg–4.5 Li–1.5 Al + 0.2 TiB alloy was performed using a DIL 805 A/D dilatometer at deformation temperatures from 250 °C to 400 °C and with strain rates of 0.01–1 s^−1^. The processing map adapted from a dynamic material model (DMM) of the as-cast alloy was developed through the superposition of the established instability map and power dissipation map. By considering the processing maps and microstructure characteristics, the processing window for the Mg–Li alloy were determined to be at the deformation temperature of 590 K–670 K and with a strain rate range of 0.01–0.02 s^−1^.

## 1. Introduction

Magnesium–lithium ultra-light alloys, as a very light structure metal, have a low density, high specific strength and stiffness, good damping performance, and excellent formability [1,2,3]. These alloys have a broad application in numerous industries, such as electronics, military, aerospace, and automotive industries [4,5,6]. It is worth noting that, with an increasing volume of Li, the deformability of the Mg–Li alloy increases; however, the strength, thermal stability, creep resistance, and corrosion significantly decrease [7,8]. The crystal structure of Mg–Li alloys can exhibit three types of microstructures caused by changes in the lithium content of the alloys. The Mg–Li phase diagram [5,9] shows that when the Li content is under ~5.7 wt.%, a hexagonal close-packed (HCP) *α(Mg)* single structure is obtained. Magnesium alloys with an Li content in the range of ~5.7–10.3 wt.%, have a duplex structure, and the eutectic structure contains an HCP phase *α(Mg)* and a body-centered cubic (BCC) phase *β(Li)*. Above 10.3 wt.%, the Li microstructure in all Mg–Li alloys is composed of a *β(Li)* phase [10]. An increase in the Li content causes a reduction in the lattice constant ratio (c/a = 1.624) of magnesium [11], as shown by Li et al. [12] with the analysis of an Mg–*x* Li–3 Al–Zn alloy, where the axial ratio c/a could be reduced from 1.624 to 1.608 when the Li fraction increased from 1 wt.% to 5 wt.%. This situation causes a reduction in critical resolved shear stresses (CRSSs) of slip systems and more slip systems being activated at ambient temperature, thus enhancing the Mg–Li deformation capacity in comparison with other Mg alloys [13]. One of the simplest and most useful techniques to enhance the mechanical characteristics of a metal’s structure is alloying or performing plastic deformation [14]. Currently, Mg–Li alloys are manufactured by thermoplastic deformation to enhance the mechanical properties of metallic structural materials to spread their application range [15]. Optimizing the forming process gives control of the formulated microstructure; therefore, it is necessary to study the deformation behavior of ultra-light Mg–Li alloys. One of the useful tools used to understand the deformation behavior of materials is the dynamic material model (DMM), which forms the basis for a processing map [8]. The processing map has been widely implemented for the characterization of numerous metals, such as Al alloys [16], Ti alloys [17], 4340 medium carbon, low-alloy steel [18], Mg alloys [19], and Cu alloys [20]. Numerous studies have been completed on the hot deformation behavior with duplex structured Mg–Li alloys; however, there is a dearth of research performed on single *α*(Mg) phase Mg–Li alloys. Li et al. [4] analyzed the hot deformation behavior of an as-extruded single *α*-phase Mg–Li–Al alloy over a temperature and strain rate range of 250–350 °C and 0.0001–1 s^−1^, respectively. Yang et al. [21] presented results on the hot deformation behavior of an as-extruded duplex structured Mg–Li–Al alloy with Sr addition and proposed the optimum restrictions of hot forming. Chen et al. [22] and Bajargan et al. [23] established the processing maps for as-extruded Mg–Li alloys modified with Ce, Y, and Zr and analyzed the microstructures in the different domains. Cheng et al. [24] examined the Mg–Sn–Zn–Al alloy and its behavior at different temperatures and hot deformation rates, and they evaluated the best hot working conditions. Askariani et al. [25] researched the hot deformation behavior of as-rolled single *α*-phase Mg–Li–Al sheets by hot compression. The microstructural and texture analysis and the deformation behavior were analyzed, and the optimized constitutive model was applied to disclose the deformation mechanism. Chen et al. [22] used the processing map technique on an as-extruded Mg–Li–Zn alloy with RE as a grain refiner over a temperature range of 250 to 450 °C and with a range of deformation rates of 1.0 × 10^−3^ to 10 s^−1^. Trojanova et al. [26] studied the deformation behavior of Mg–Li–Al alloys in the as-cast state at temperatures up to 250 °C. Establishing a constitutive model is a popular and essential way to characterize the deformation behavior of metal structured materials at various strains, strain rates, and temperatures. An Arrhenius-type constitutive model has been extensively applied for numerous different metals, not just in the characterization of ultra-light Mg–Li alloys. Wu et al. [27] formed an Arrhenius-type constitutive model for a high-strength Ni–Cr–Mo–V low-alloy steel. The proposed constitutive model offered a prediction of the flow stress with *R* = 0.977.

In this work, hot compression tests on single α-phase as-cast Mg–4.5 Li–1.5 Al alloy with an HCP structure and with TiB addition as a grain refiner to further improve the strength were used to systematically analyze the deformation behavior of the alloy at elevated temperatures. The optimized constitutive model and hot working terms of the Mg–Li alloy with TiB were established.

## 2. Materials and Methods

The as-cast Mg–4.5 Li–1.5 Al + 0.2 TiB alloy was used throughout this work. The applied magnesium–lithium alloy had a single *α*-Mg phase, according to the binary diagram of Mg–Li elements [6]. Commercial pure magnesium ingots (min. 99.5%), lithium strips (99.9%), aluminum ingots (99.98%), and TiB rods were added to a mild steel crucible located in an induction heater type VSG 02 manufactured by the Balzers company (Bremen, Germany) [28]. Firstly, the high-purity Mg ingots were smelted in a steel crucible under a protective argon atmosphere, at a pressure of 650 torr and a temperature of 700–720 °C, until a melt was formed. In the next step, the prepared liquid metal was cast into the cold graphite mold into rod-shaped ingots with dimensions of ø20 × 100 mm. Afterward, cylindrical samples with a diameter of 5 mm and a length of 10 mm were machined. The analysis of the chemical composition of the prepared ultra-light Mg–Li alloy was determined using inductively coupled optical emission spectrometer with an inductively coupled plasma (ICP-OES). The chemical composition of the studied alloy used in the experiment is listed in Table 1.

**Figure 1:**
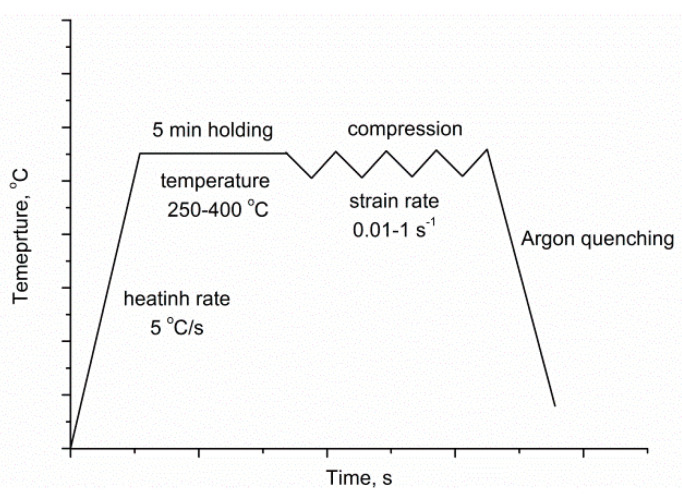
The experimental scheme for the hot simulation.

Analysis of elements in the alloy was performed using an inductively coupled optical emission spectrometer with an inductively coupled plasma (ICP OES) OPTIMA 5300V, made by PerkinElmer (Waltham, MA, USA). The uniaxial hot compression experiments were completed using a DIL 805 A/D dilatometer delivered by TA Instruments (Zaventem, Belgium) [29], over a strain rate range of 0.01 s^−1^ to 1 s^−1^ and a temperature range of 250 °C to 400 °C, using a PtRh10–Pt thermocouple rod under an argon protective atmosphere to prevent sample oxidation. Before the compression experiments, the samples were heated up to the deformation temperature at a heating rate of 5 °C·s^−1^ and held isothermally for 5 min, before being compressed at a constant strain rate. The experimental scheme for the hot simulation is given in Figure 1. The deformation degree was 60%, corresponding to a true strain of approximately 1. After the hot deformation experiment, high-flow argon quenching was carried out to preserve the deformed microstructure. For metallographic characterization, the compressive cross-section was prepared from the same place of the specimen. A Leica microscope (Vienna, Austria) with Q-WinTM image analyzer software (ver. 4.8.2.0) was used for microstructure observation. The average grain size was measured according to the ASTM E112-13 standard test methods. The fresh etchant used in the experiment was composed of 1 mL of HNO_3_, 24 mL of water, and 75 mL of ethylene glycol. The phase structure was detected using an X’Pert diffractometer (Almelo, The Netherlands). The analysis of the obtained diffraction patterns was made in the Panalytical High Score Plus software (Version3.0e), containing a dedicated file base of PAN-ICSD phase identification. For X-ray diffraction characterization, a Co target, scan rate of 0.03 step/s, and a scan 2θ range of 30° to 110° were applied.

## 3. Results and Discussion

**Figure 2:**
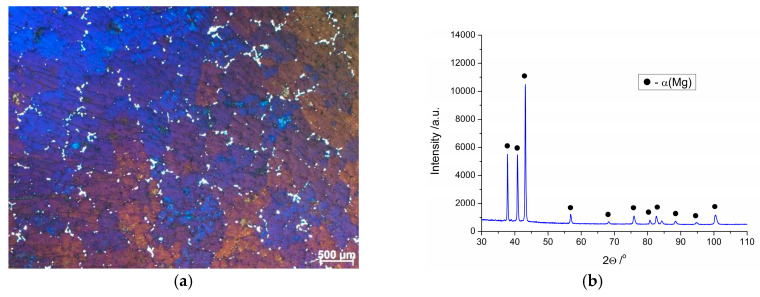
(**a**) LOM image and (**b**) XRD pattern of the as-cast Mg–4.5 Li–1.5 Al + 0.2 TiB alloy.

Figure 2 presents the microstructure of the as-cast Mg–4.5 Li–1.5 Al + 0.2 TiB alloy and its XRD pattern. Our previous work [28] reported that Mg–4.5 Li–1.5 Al + 0.2 TiB alloy is composed of an HCP lattice structure *α(Mg)* phase. A small amount of the *η(LiAl)* intermetallic element with a B2 structure heterogeneously distributed along the phase boundaries could be found in the microstructure (Figure 2a). The average grain size of the analyzed alloy in the as-cast state was approximately 520 μm. Diffraction peaks coming from the *α(Mg)* phase could only be identified in the XRD (Figure 2b) pattern due to the low content of compounds. The identified crystalline phase was described on the basis of card no. 98-016-2414 (PAN-ICSD). Hence, in the presented work, the influence of precipitates throughout the hot compression experiment of the analyzed alloy can be disregarded. Additionally, high peaks at 2θ of 39°, 41°, and 43° can be seen, and the intensity variation of these peaks was tiny, meaning that there was no noticeable texture in the analyzed structure.

**Figure 3:**
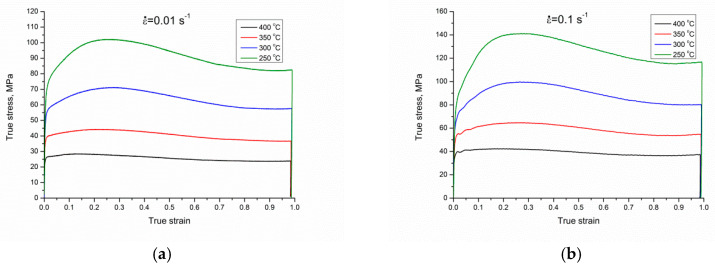

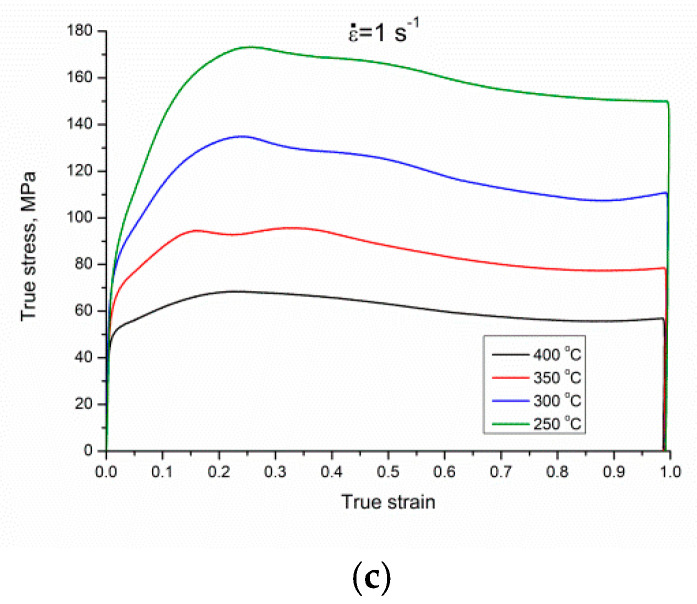
The true stress–strain curves of the Mg–4.5 Li–1.5 Al + 0.2 TiB alloy during hot compression tests at varying strain rates: (**a**) 0.01 s^−1^; (**b**) 0.1 s^−1^; (**c**) 1 s^−1^.

The true stress–strain curves of the Mg–4.5 Li–1.5 Al + 0.2 TiB alloy in different conditions are presented in Figure 3. From the true stress–strain curves, it can be stated that the flow stress value is dependent on the strain rates and deformation temperatures. It can be observed that with a decrease in strain rates or an increase in the deformation temperature, the peak stress slowly decreased. Moreover, it can be noted that, while the strain rate was constant, flow stress grew during the first stage. Afterward, the stress rate decreased until a maximum stress was obtained, with a gradual reduction until it smoothed out once again. Nearly all of the curves manifested an evident single-peak event. In the first stage of alloy deformation, the dislocation multiplication rate was fast, correspondingly occurring during the rapid development of dislocation density. Hence, the stress increased, and the work hardening process was principal [30]. However, when the strain grew to a specific point, the storage energy of deformation increased, attended by dynamic softening, but work hardening was still the primary deformation phenomenon. The shape of the true stress–strain graph dropped and the graph became smooth and flat [31,32,33]. After passing the peak stress, the graphs showed a constant drop due to the presence of dynamic softening in the form of dynamic recrystallization (DRX) and dynamic recovery (DRV). After stability within the work hardening and dynamic softening was achieved, the flow stress became constant. This event indicated a weak and robust alternation of work hardening and dynamic softening, caused by the iteration of annihilation and dislocation accumulation. To gain knowledge about the correlation of the different indicators and flow stress, and to monitor the high-temperature plastic deformation behavior of the alloy, an Arrhenius criterion was employed to define the correlation within the flow stress and multiple indicators, where a constitutive model of $\dot{\varepsilon }$ could be estimated as follows [34]:

(1) $$\dot{\varepsilon }=A{\left[sinh\left(\alpha \sigma \right)\right]}^{n}\exp\left(-\frac{Q}{RT}\right).$$

Furthermore, the influences of strain rate and temperature on the deformation behavior could be further denoted by the Zener-Hollomon (*Z*) parameter using an exponent-type equation.

(2) $$Z=\dot{\varepsilon }\exp\left(\frac{Q}{RT}\right)=A{\left[sinh\left(\alpha \sigma \right)\right]}^{n},$$

where $\dot{\varepsilon }$ is the strain rate (s^−1^), *Q* is the activation energy for hot deformation (kJ/mol), *σ* represents the flow stress (MPa), *R* represents the universal gas constant (mol^−1^·K^−1^), *T* is the thermodynamic temperature (K), and *n* is the stress exponent, whilst *A*, *n* (stress exponent), and *α* (stress coefficient) are constants. The correlation within the *Z* parameter and *r* could be estimated as follows [35]:

(3) $$Z=\dot{\varepsilon }\exp\left(\frac{Q}{RT}\right)=A_{1}\sigma^{n_{1}}.$$

(4) $$Z=\dot{\varepsilon }\exp\left(\frac{Q}{RT}\right)=A_{2}\exp\left(\beta \sigma \right).$$

Equation (2) is appropriate for every level of stress, Equation (3) can be applied in low-stress conditions (*ασ* < 0.8), and Equation (4) is suitable for high-stress conditions (*ασ* < 1.2), where *n*_1_ and *b* are material constants. The stress index *α* could be described as follows [24]:

(5) $$\alpha =\frac{\beta }{n_{1}}.$$

To rationalize these equations, we used the natural logarithms of Equations (3) and (4), giving

(6) $$ln\dot{\varepsilon }=n_{1}ln\sigma +lnA_{1}-\frac{Q}{RT},$$

(7) $$ln\dot{\varepsilon }=\beta \sigma +lnA_{2}-\frac{Q}{RT}.$$

**Figure 4:**
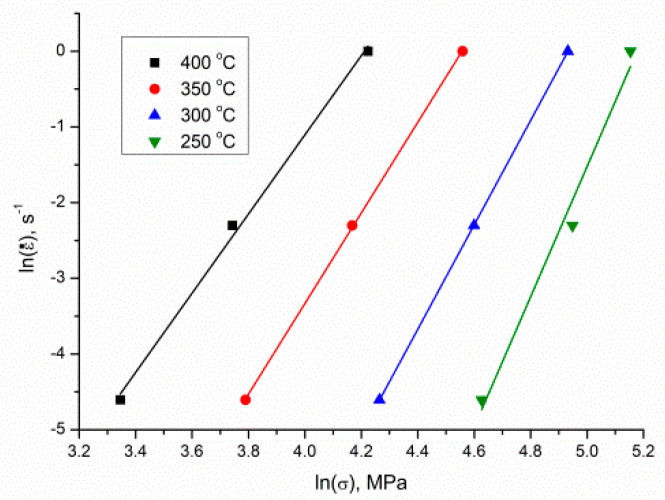
The relationship between *ln*($\dot{\varepsilon }$) and ln(*σ*).

**Figure 5:**
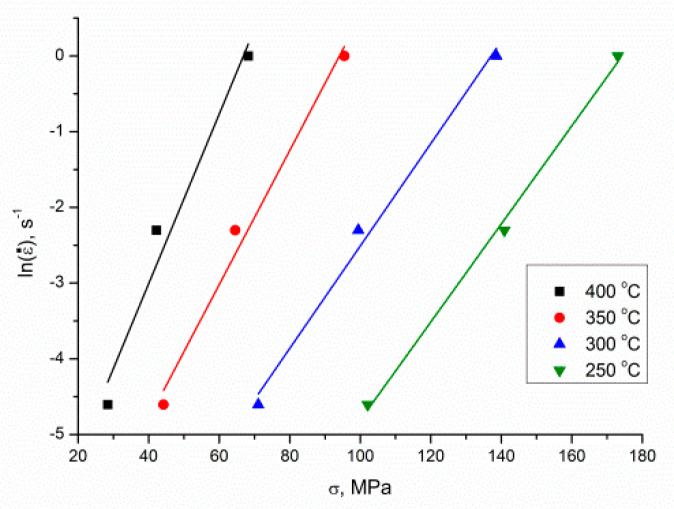
The relationship between *ln*($\dot{\varepsilon }$) and *σ*.

Using the peak stress, *σ*, following different deformation conditions, and utilizing a least-square method to implement linear regression, the mean value of *n*_1_ and *β* could be calculated, i.e., 6.6777275 and 0.08323 MPa^−1^, respectively, from the graphs of $ln\dot{\varepsilon }\;\mathrm{vs}.\;ln\sigma$ (Figure 4) and $ln\dot{\varepsilon }\;\mathrm{vs}.\;\sigma$ (Figure 5); accordingly, $\alpha =\frac{\beta }{n_{1}}=0.013384429$ MPa^−1^.

Using the natural logarithm from both sides of Equation (1), Equation (8) could be obtained.

(8) $$ln\dot{\varepsilon }=nln\left[sinh\left(\alpha \sigma \right)\right]+lnA-\frac{Q}{RT}.$$

**Figure 6:**
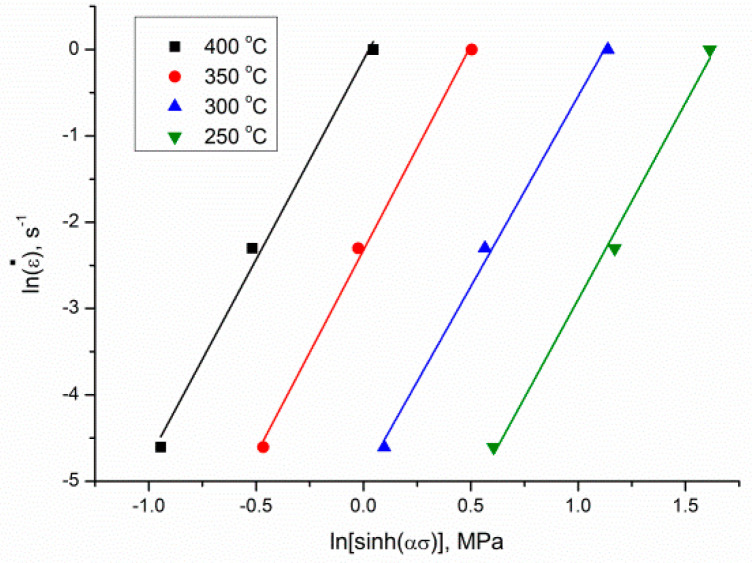
The relationship between *ln*($\dot{\varepsilon }$) and ln[sinh(ασ)].

By replacing *α*, the peak stress *σ* followed different deformation states and $\dot{\varepsilon }$, and by applying the least square to execute the linear regression, the stress exponent *n* could be calculated as 4.58128 from graphs $\dot{ln\varepsilon }$ and ln[sinh(ασ)] given in Figure 6. To get the *Q* value, Equation (8) was transformed as follows [30]:

(9) $$Q=RnS=R{\left\{\frac{\partial (ln\dot{\varepsilon )}}{\partial ln\left[sinh\left(\alpha \sigma \right)\right]}\right\}}_{T}{\left\{\frac{\partial ln\left[sinh\left(\alpha \sigma \right)\right]}{\partial \left(\frac{1}{T}\right)}\right\}}_{\dot{\varepsilon }}.$$

**Figure 7:**
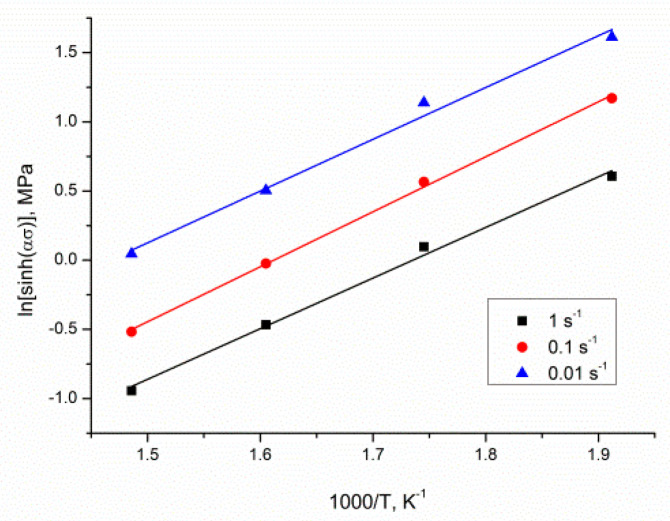
The relationship between ln[sinh(ασ)] and temperature (1000/T, K^−1^).

The mean value of *s* could be specified from the graph using ln[sinh(ασ)]−1000/T (Figure 7), giving a value of 3.79199.

Following Equation (9), the deformation activation energy of Mg–4.5 Li–1.5 Al + 0.2 TiB *Q* could be determined as 144.4322043 kJ/mol. The calculated value was lower than those of the rolled single α-phase Mg–Li alloy (211 kJ/mol) [24], as-cast LAZ532 (160 kJ/mol) [3], commercial AZ80 alloy (216 kJ/mol) [36], extruded-state *α(Mg)–β(Li)* duplex phase Mg–Li alloy (148 kJ/mol) [37], as-cast Mg–2 Zn–0.3 Zr–0.9 Y alloy (236.2 kJ/mol) [38], and as-cast Mg–3 Sn–Ca alloy (236 kJ/mol) [39]. Despite being related to the other Mg–Li alloys with *α + β* duplex phases or a single *β*-phase, the deformation activation energy in the presented work was higher than those of the as-cast *α + β* alloy (127 kJ/mol) [40], the as-cast single *β*-phase Mg–Li alloy (95 kJ/mol) [41], the as-cast Mg–8 Li–3 Al–2 Zn alloy modified with Zr (108 kJ/mol) [8], the as-cast Mg–9 Li–1 Zn alloy (127 kJ/mol) [42], the as-cast Mg–11.5 Li–1.5 Al alloy (95 kJ/mol) [43], the as-cast Mg–3 Sn–2 Al–1 Zn–5 Li (139 kJ/mol) [44,45], the as-cast Mg–9 Li–3 Al alloy with Sr addition (110 kJ/mol) [46], and as-cast LA43M (110 kJ/mol) [4].

Research [47] showed that, at 135 kJ/mol, the lattice self-diffusion activation energy of magnesium takes place, and, at the 92 kJ/mol grain boundary, diffusion activation energy takes place. In the presented work, with the addition of Li as an alloying element, the axial ratio (c/a) decreased, causing an increase in the activity of the non-basal slips and a reduction in activation energy *Q* [48,49].

**Figure 8:**
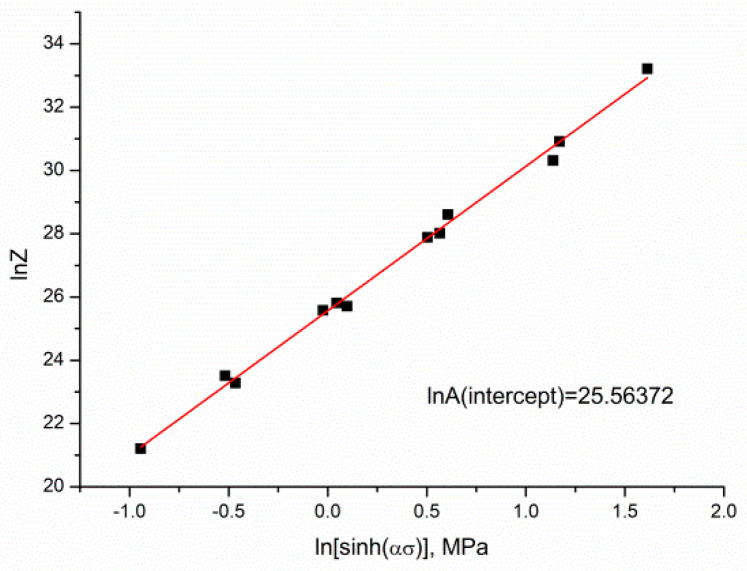
The relationship between *lnZ* and ln[sinh(ασ)].

A linear relationship between *lnZ* and *ln[sinh(ασ)]* in Mg–4.5 Li–1.5 Al + 0.2 TiB can be observed in Figure 8. The intercept of the fitted curve (*lnA*) was 25.56372 and A was 1.265268 × 10^11^.

Conclusively, adding the values of *α*, *A*, *n*, and *Q* into Equation (2), a constitutive model for the analyzed Mg–4.5 Li–1.5 Al + 0.2 TiB alloy could be proposed.

(10) $$\dot{\varepsilon }\;=\;1.265268\times 10^{11}{\left[sinh\left(0.013384429\sigma \right)\right]}^{4.58128}\exp\left(-\frac{144432}{RT}\right).$$

Moreover, the linear regression statistics of the results are presented in Table 2.

### The Processing Maps

From the dynamic material model, a processing map could be used to analyze the hot workability of the Mg–Li alloy, focusing on the power dissipation and instability maps. In the processing map, the efficiency of power dissipation (*η*), on the basis of the values of the flow stress at different states of deformation at a true strain of 0.3 and 0.6, was estimated, as proposed by Prasad and Seshacharyulu [50,51].

(11) $$\eta =\frac{2m}{2m+1}.$$

The continuum instability (*ξ*) model was represented as follows:

(12) $$\xi \left(\dot{\varepsilon }\right)\;=\;\frac{\partial lg\left(\frac{m}{m+1}\right)}{\partial lg\dot{\varepsilon }}+m\leq 0.$$

**Figure 9:**
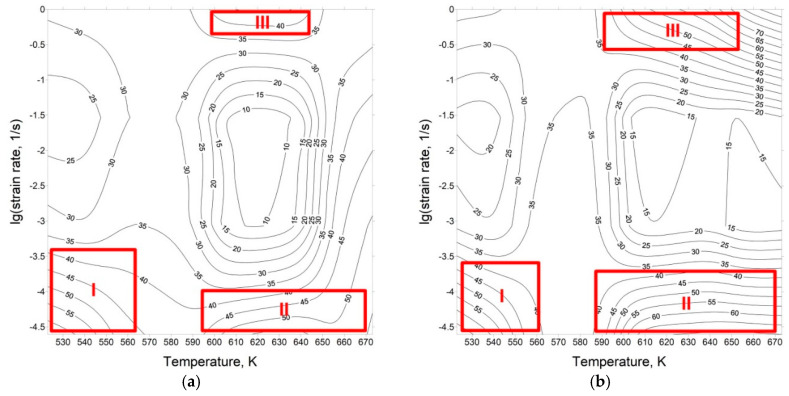
The processing maps of the Mg–4.5 Li–1.5 Al + 0.2 TiB alloy at a strain of (**a**) 0.3 and (**b**) 0.6; the contour numbers indicate the percentage efficiency of power dissipation (*η*%).

The DMM law demands that (1) the deformation of a material is a nonlinear energy dissipation body, and (2) the total energy (P) consumed by the material may be classified into two parts: the first section is the dissipation energy (G content) which is applied to generate plastic deformation, and the second section is described as complementary dissipation energy (J co-content) which is related to the obtain material microstructure progression. The distribution relationship between *J*, the co-content, and the *G* content is determined by the strain rate sensitivity *m*, which could be defined as follows:

(13) $$m=\frac{\partial J}{\partial G}=\frac{\dot{\varepsilon }\partial \sigma }{\sigma \partial \dot{\varepsilon }}=\frac{\partial ln\sigma }{\partial ln\dot{\varepsilon }},$$

where $m=\frac{\partial \left(lg\sigma \right)}{\partial lg\dot{\varepsilon }}=\frac{1}{n}$ is the strain rate sensitivity. The processing map could be graphed as contour maps by projecting the *η* and *ξ* maps, within the related parameters of deformation temperature and strain rate, as presented in Figure 9.

By using cubic spline interpolation, an estimation of flow stresses at various strain rates and temperatures, the efficiency of *η*, and the instability indicators under multiple states were calculated. Figure 9 represents the processing maps related to the true strain of 0.3 (peak stress) and 0.6. It is worth mentioning that the flow instability regions took place when ξ was negative, causing the occurrence of defects such as intercrystalline cracking and cave formation. Studies of the processing maps showed that *ξ* was positive under each test state, which indicates that there were no instability zones. Therefore, the hot workability of Mg–4.5 Li–1.5 Al + 0.2 TiB was good for all tested conditions.

According to the processing maps, the three domains with peak efficiency over 40% were Domain I which occurred within 520–560 K and strain rate range of 0.01–0.03 s^−1^. The second domain appeared at a relatively low strain rate over a range of 0.01–0.02 s^−1^ and in the high temperature range of 590–670 K. For Mg alloys with low stacking fault energy, the dynamic recovery appears when *η* < 30%, while dynamic recrystallization occurs when *η* > 30% and may increase in the thermal processing efficiency with its softening, thereby stabilizing the flow [52]. In the analyzed zone, atomic diffusion was accelerated by the increased deformation temperature, facilitating DRX, which increased the driving force to flow softening. Moreover, the formation of the small grains by DRX was provided by the low strain rate and extended development time, leading to a more significant processing result. The third domain occurred in the temperature and strain rate range of 600–650 K and 0.65–1 s^−1^, respectively. In general, the formation of fine equiaxed grains is defined by a high value of *η*, which relates to good processability of the processed material. An accurate processing range can be selected on the basis of zones that are characterized with a low *η* and are without instability regions.

In the presented work, the stability regions with a high power dissipation factor *η*, which relates to the optimal treatment states of DRX and DRV, were less productive when *η* < 50% because the generation of interfaces formulated by dislocation generation and simultaneous recovery took the energy. Conversely, the occurrence of cracking processes was characterized when *η* > 50%, because the energy conversion onto the surface was more effective [29].

**Figure 10:**
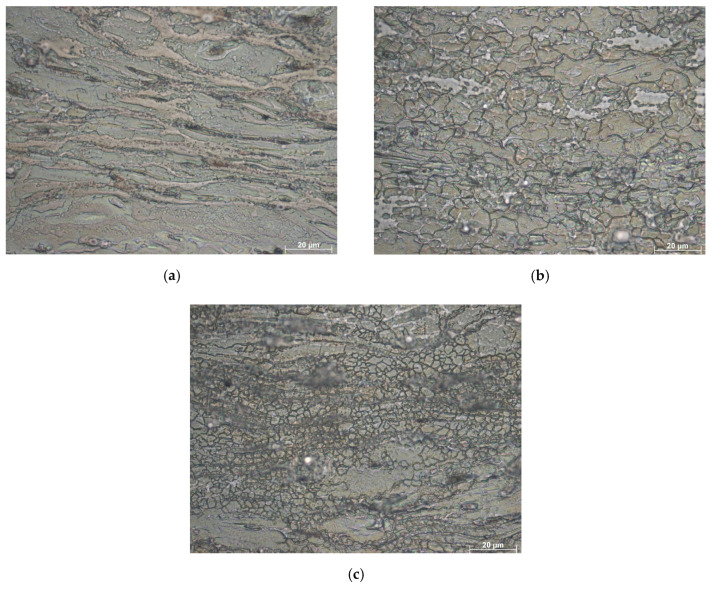
The microstructures of the Mg–4.5 Li–1.5 Al + 0.2 TiB at different deformation strain rates and temperatures: (**a**) 250 °C/0.01 s^−1^, (**b**) 350 °C/0.01 s^−1^, and (**c**) 350 °C/1 s^−1^.

The compressive hot deformation study of as-cast LA43M alloy [4] provides information about similar regions in the processing maps, which are indicative of high-power dissipation capability. To confirm this interpretation for the three high-efficiency zones determined on the processing maps of the analyzed ultralight Mg–Li alloy, microstructural observations were carried out. Figure 10 represents the LOM images of the components under hot compression tests at 250 °C/0.01 s^−1^, 350 °C/0.01 s^−1^, and 350 °C/1 s^−1^ at a true strain of 1, related to the microstructure morphology in the flow stability zones in the processing map. Changes in the microstructure of the analyzed alloys were a result of the flow stress correlated to the deformation temperature and strain rate, and they indicated a constitutive model analysis and thermal processing map of the Mg–4.5 Li–1.5 Al + 0.2 TiB alloy. Figure 10a represents the deformed microstructure, to a slender shape, at 250 °C/0.01 s^−1^, related to the first high-efficiency zone.

After deformation, in the observed regions, the grain boundary exhibited a necklace-like dynamic recrystallization structure after hot deformation.

During the hot compression experiment, in the analyzed microstructure, some initial un-DRXed grains developed in the elongated shape longitudinal to the direction of deformation; however, many refined DRXed grains formed in the structure. The differences in mechanical properties of the studied material were a result of the appearance of the necklace-like structure [36]. Figure 10b represents the microstructure after a deformation test at 350 °C/0.01 s^−1^ that corresponds to the high-efficiency Domain II region. At this deformation state, complete DRX grains occurred, and a fine and homogeneous microstructure morphology could be observed. The rapid grain development in the Mg–Li alloy was caused by an increased temperature to 350 °C. Moreover, the low strain rate ensured fine grains formed by DRX with more time to develop, resulting in an improved processing effect.

Figure 10c represents the microstructure of the alloy after the hot deformation test with a deformation temperature of 350 °C and the highest applied experiment strain rate of 1 s^−1^. The DRX grains were observed in the analyzed sample; however, when the strain rate decreased to 0.01 s^−1^, as given in Figure 10b, the size of the DRX grains gradually increased. This phenomenon confirmed that the reduced strain rate benefits the generation of DRX grains.

## 4. Conclusions

In this study, uniaxial hot compression experiments of as-cast Mg–4.5 Li–1.5 Al alloy modified with TiB were completed over a temperature range of 250–400 °C and a strain rate range of 0.01–1 s^−1^. The main conclusions are as follows:

1. The stress–strain curves of the as-cast Mg–4.5 Li–1.5 Al + 0.2 TiB alloy under thermal compression were characterized by dynamic softening. When the strain increased, the work hardening effect was more efficient. The true stress decreased with an increase in the temperature at the same strain rate. Moreover, the peak stress decreased with a decrease in the strain rate at the same temperature.
2. The activation energy, *Q*, of the analyzed alloy was 144.34 kJ/mol. The correlation among flow stress, deformation temperature, and strain rate was calculated using an Arrhenius-type constitutive equation with a hyperbolic sine function,
  $$\dot{\varepsilon }=1.265268\times 10^{11}{\left[sinh\left(0.013384429\sigma \right)\right]}^{4.58128}\exp\left(-\frac{144432}{RT}\right).$$
3. According to the dynamic material model, the processing maps at a strain of 0.3 and 0.6 were established, and the stable regions were determined. Generally, the stability domains of the analyzed alloy occurred in the low-strain-rate region. Moreover, processing maps indicated through calculations that *ξ* was positive under all test conditions, suggesting that there were no instability regions.
4. The processing maps at a true strain of 1 revealed three stable domains. The complete processing map and microstructure study showed that the best parameters of processing were established to be over a deformation temperature range of 590–670 K and a strain rate range of 0.01–0.02 s^−1^.

## Figures and Tables

**Table 1:** The chemical composition of the alloy (wt.%).

Li	Al	Si	B	Ti	Fe	Mg
4.66	1.80	0.0052	0.0019	0.0069	0.0019	Balance

**Table 2:** Statistics for the linear regression between *ln*$\dot{\varepsilon }$ and *σ* for the analyzed Mg–4.5 Li–1.5 Al + 0.2 TiB alloy.

Deformation Temperature (°C)	$\ln\dot{\varepsilon }$–*lnσ*		$\ln\dot{\varepsilon }$–*σ*		$\ln\dot{\varepsilon }$–*ln*[*sinh*(*ασ*]	
	Regression Equation	Correlation Coefficient	Regression Equation	Correlation Coefficient	Regression Equation	Correlation Coefficient
250	y = 8.59*x* − 44.50	0.99196	*y* = 0.06*x* − 11.28	0.99854	*y* = 4.55*x* − 7.44	0.99764
300	y = 6.89*x* − 34.02	0.9999	*y* = 0.06*x* − 9.27	0.99574	*y* = 4.41*x* − 4.95	0.99834
350	y = 5.98*x* − 27.27	0.9998	*y* = 0.08*x* − 8.33	0.99302	*y* = 4.733*x* − 2.32	0.99869
400	*y* = 5.23*x* − 22.03	0.99849	*y* = 0.11*x* − 7.48	0.98478	*y* = 4.62*x* − 0.11	0.99679
